# Good practices in harnessing social media for scholarly discourse, knowledge translation, and education

**DOI:** 10.1007/s40037-020-00613-0

**Published:** 2020-08-20

**Authors:** Daniel Lu, Brandon Ruan, Mark Lee, Yusuf Yilmaz, Teresa M. Chan

**Affiliations:** 1 Department of Psychiatry, Faculty of Medicine, University of British Columbia, Vancouver, BC Canada; 2 McMaster Education Research, Innovation, and Theory (MERIT) unit, McMaster University, Hamilton, Ontario Canada; 3 Department of Medical Education, Faculty of Medicine, Ege University, Izmir, Turkey; 4 Program for Faculty Development, Office of Continuing Professional Development for the Faculty of Health Sciences, McMaster University, Hamilton, Ontario Canada; 5 Department of Medicine, Division of Education & Innovation and Division of Emergency Medicine, Faculty of Health Sciences, McMaster University, Hamilton, Ontario Canada

**Keywords:** Social media, Knowledge Translation, Education, Scholarly Discourse

## Abstract

**Introduction:**

There still remains a gap between those who conduct science and those who engage in educating others about health sciences through various forms of social media. Few empirical studies have sought to define useful practices for engaging in social media for academic use in the health professions. Given the increasing importance of these platforms, we sought to define good practices and potential pitfalls with help of those respected for their work in this new field.

**Methods:**

We conducted a qualitative study, guided by constructivist grounded theory principles, of 17 emerging experts in the field of academic social media. We engaged in a snowball sampling technique and conducted a series of semi-structured interviews. The analytic team consisted of a diverse group of researchers with a range of experience in social media.

**Results:**

Understanding the strengths of various platforms was deemed to be of critical importance across all the participants. Key to building online engagement were the following: 1) Culture-building strategies; 2) Tailoring the message; 3) Responsiveness; and 4) Heeding rules of online engagement. Several points of caution were noted within our participants’ interviews. These were grouped into caveat emptor and the need for critical appraisal, and common pitfalls when broadcasting one’s self.

**Discussion:**

Our participants were able to share a number of key practices that are central to developing and sharing educational content via social media. The findings from the study may guide future practitioners seeking to enter the space. These good practices support professionals for effective engagement and knowledge translation without being harmed.

**Electronic supplementary material:**

The online version of this article (10.1007/s40037-020-00613-0) contains supplementary material, which is available to authorized users.

## Introduction

Social media is now a dominant medium for discourse, debate and education. Recently, the COVID-19 pandemic has highlighted how crucial social media has become for both our daily and professional lives [1, 2]. With the staggering explosion of content online both within health professions education and knowledge translation [3], we are entering into an *attention economy* for learners within the increasingly crowded social media space [4]. Within business, marketing professionals manage their brand’s attention in a vast sea of attention-seeking stimuli. In the online world of health professions education, a similar attention economy is emerging; teachers and scientists are deploying many different tactics to engage their intended audiences.

One of the great challenges in engaging scientists and investigators (including those in health professions education) in social media activities is their perceptions of its usefulness. Some individuals who have spent their lives devoted to generating science may not feel adequately trained to engage in social media-based techniques for disseminating their scholarship [5, 6] or sufficiently rewarded by traditional tenure and promotion processes [7]. These factors have contributed to social media’s under-utilization by the scientific community despite it having shown to have direct implications for enhancing visibility of science [8, 9].

In a recent scoping review, while there was an abundance of descriptive studies (*n* = 242) and conceptual pieces (*n* = 192), there were very few clarification studies (*n* = 5) about the usage of social media for education and/or knowledge translation [3]. None of these studies attempted to define good practices used by experienced providers. We sought opinions from those respected for their academic social media work to generate a list of good practices and potential pitfalls.

## Methods

We conducted a constructivist grounded theory study to determine the good practices and potential pitfalls observed by experts in the areas of social media education and knowledge translation.

### Sampling

We engaged in a snowball sampling technique, which has been used in the field of social media research within health professions education since it is an evolving field with rapidly changing techniques and protocols [6, 10–12]. We initially randomly drew from a previously published list of social media influencers within emergency medicine [12], since this field has been shown to be quite active in social media scholarship and publications, according to a recent review [2]. However, since there has been a marked adoption of social media across all sectors since this original list, we employed a snowball sampling technique as the expertise in this area is not fixed and is evolving. As such, snowball sampling allowed our interviewees to then further nominate individuals whom they admired as experts in one of the following areas: 1) Knowledge translation and teaching; 2) Acting as an interactive scientist or investigator; 3) Engaging as a critical clinician [5]. Individuals were initially contacted by email or social media to engage in our study. We attempted to sample across all three groups.

### Context

The context of the study was the digital community of social media knowledge translation specialists and educationalists. Although we began our study of social media experts using a social media influencers list from one specific specialty (emergency medicine), our context was broader due to our snowball sampling.

### Ethics

Our team received ethical approval from the Hamilton Integrated Research Ethics Board (# HIREB-5609).

### Data collection methods

A series of semi-structured interviews were conducted by our team’s research assistants (BR, AM). The research assistants were initially trained via simulation and practice with feedback. Initial transcripts for research assistants were also reviewed initially by the primary investigator (TC) to provide insights for further topic exploration as part of our constant comparative analysis. Each interview was conducted using Zoom (Zoom Video Communications, Inc., San Jose, CA, USA) with audio capture on our local computer. The interview guide is found in the Electronic Supplemental Materials (Appendix 1).

### Data processing

The audio files were sent to a trained and experienced medical transcriptionist, who generated written transcripts from the audio files. Participants were assigned a gender-matched alias. The transcripts were then verified or corrected as needed by the interviewer and investigatory team to ensure the accuracy of the transcript.

### Data analysis

We conducted our analysis using a constant comparative method, iteratively delineating a series of codes aligned with various good practices and potential pitfalls throughout our coding process. Our analysis team (BR, DL, TC) met multiple times over a number of months, analyzing transcripts for relevant themes after batches of 2–4 interviews were completed. Each coding session, a code book was updated, with relevant codes being organized and reorganized until we reached thematic sufficiency within our dataset about good practices and potential pitfalls to avoid. These analysis sessions allowed us to iteratively refine the prompts or sub-prompts used by our interviewer, guiding us better towards sufficiency.

### Sensitization

In constructivist grounded theory, researchers may be *sensitized* by concepts that have preceded their present work. These are concepts that inform their analysis and are acknowledged fully for the readership. For our analysis we were sensitized by two concepts: 1) Davenport and Beck’s *attention economy* [4]; and 2) The new types of social media scholars (translational teachers, interactive investigators, and critical clinicians), which was a conceptual framework that had been previously proposed in the literature [5].

The concept of the *attention economy* comes from a concept in the business world which highlights the increasing limitations of end-user (or customer) attention as a new type of economic driver; specifically, human attention is now a form of currency due to limitations in its supply, thereby forcing those who demand our attention to compete. Davenport and Beck’s work was mainly used for the analysis portion of our study. When reading transcripts, this concept helped us to detect practices that were more akin to business structures or marketing strategies that our experts were employing for the purposes of disseminating education. Their work describes several key concepts within the attention economy: *voluntary attention, attractive attention, aversive attention, front-of-mind attention,* and *back-of-mind attention.* Moreover, they describe the concept of *attention management*, a task that seems to resonate with educators.

The other concept of the new types of social media scholars, which emerged due to the increasing use of social media for knowledge translation, has been highlighted because our principal investigator (TC) was a lead author on this conceptual work, and its influence is undeniable in this present study—we framed our recruitment and interview guide around these types of scholars, specifically seeking out those who fit within this framework.

### Techniques to enhance rigor and trustworthiness

To ensure rigor of our analysis, we engaged two members of our research team (ML, YY) to conduct an audit of our analysis trail. They were given full access to primary transcripts and the final codebook. The plan for resolving conflicts at this stage was to engage in discussions around areas of concerns. Consensus building techniques were used to resolve any issues that arose.

### Reporting

This report adheres to the Standards for Reporting Qualitative Research reporting guidelines [13].

## Results

### Demographics of participants

In total, 17 individuals were interviewed. See Tab. 1 of the online Supplementary Material for details about key demographics. Social media platforms used by our participants can be found in Tab. 2 of the online Supplementary Materials, which is also online. The interviews were on average 30.6 min long, ranging from 18.6 to 52.1 min. This yielded a total of 189 pages of transcripts. Within this group, nine individuals self-identified as translational teachers, five individuals identified as critical clinicians, and three saw themselves as interactive investigators.

**Table 1:** Best usages for various social media platforms

Platform	Best practices	Examples mentioned
Twitter	Users may find it prudent to divide out different accounts for different usages. Some suggested divisions: – Person-level professional account – Group/Institutional – Research Team	Group/Institution: @WeAreCanadiEM (www.canadiem.org) Residency Program account Departmental account
		Research Team: @METRIQstudy (www.metriqstudy.org)
	When engaging in social media promotion of research, consider the following practices when generating a tweet: – Include an image in the tweet – Use descriptive language – Tag people involved – Tagging related organizations or granting agencies involved in the work – Tagging the journal that the article was published within – Using hashtags to join the right conversation Advanced concepts include: – Tweet chats	Understand the nuances between accounts. Have a clear intent and purpose for each account
		“*You have to be aware of what the purpose of each account is and certainly the purpose of my department’s account is very different than my account*.”—Piper
		“*I am really deliberate in my use of hashtags. I also try not to spam. (Laughing). So, like three or less hashtags in a tweet… Also, in my communication, I will take tag certain people that I want to make sure that they are aware.*”—Grace
Facebook	Person-level account	N/A
	Facebook pages—Group/Institutional	CanadiEM Facebook Page
Instagram	Group/Institutional	PEM Morsels CanadiEM
Closed social platform	Used for within team communication to enhance the functioning of a team of social media users or producers (e.g. blog community)	Groups using Slack: ALiEM CanadiEM
Blog	Used for housing general summaries and disseminative works, but also to release new scholarly contributions via a digital platform.	*“… we have been producing a case of the week. And disseminating that internationally with our pathology residents and fellows … using blogging platform to do that with the question. It is a short snippet of the case—80 words or less. It has an image or digital image. Like a digital scan and pathology slide as well as the question that goes with it. So that is another way that we have used social media for learners and also for our faculty.”—Grace*
Podcasts	A possible outlet for digital scholarship and academic output. Can be used as its own free-standing academic output, since it is seen as digital scholarship	EM Basic (for junior trainees) EM Guidewire (involves residents)
Other platforms mentioned without good practice advice: Reddit, Google Plus, LinkedIn, Blogs, Read by QX, ResearchGate

**Table 2:** Good practices for engagement online

Good practice	Explanatory quote
Use common sense	“I guess it is fairly straightforward. Just don’t be an idiot… I don’t know I guess I don’t do heaps and heaps of tweeting myself but if I am responding to somebody it will usually be to make sure that I say something positive or say nothing at all.”—Sheila
Clearly identifying yourself, including conflicts of interest	“I clearly identify myself as my Twitter handle is not my name, but my name is on my Twitter profile. And yeah, I think that is pretty straightforward… like, just behave properly.”—Sheila
	“It’s just really thinking about your profile is a best practice. Just thinking about being transparent to the community [about] who you are and … what you are going to be communicating about in that social media platform. So, [regarding] your presence in your profile, I think another best practice that I really try and think about and encourage other people to think about as well.”—Grace
Aligned with self and institution	“I’ve tried to make all of my intentions honorable and things that I would be proud of representing and that would reflect on my institution and institutions in a positive way. And so, my interactions again are founded on what is going to be best for patient care and kindness and making my intentions honorable. And so those are all things that I think of as core values that the institutions that I am affiliated with … support.”—Edward
Understand the intention of each account in each platform	“I am a big believer in aligning my technology with my goals that I want to achieve. And also separating personal and professional. So, I chose Twitter because at the time it was where I was connecting with people in medical education, finding that it seems like that is where the audience that I wanted to connect with professionally was currently at. I felt like Facebook was more personal. Um, and that Instagram and other, and Instagram especially I guess was just starting to emerge when I was working with getting myself established in medical education. Um, now have I moved to Instagram. I use Instagram, um, more to help I guess personal[ly], but I guess some I had done some work connecting with other professionals on it just a little. Slack is one that I use …”—Grace
Maintaining respect	“I think in general you try to um, be polite and professional. Like I don’t necessarily think delving into in depth articles on Twitter is necessary or appropriate, um, however responding to people who are having questions or being critical of things I think it is a very reasonable way to go. And [I] try to do it in a respectful way… And that can be productive [in] conversation”—Trevor
Stay positive	“if I am responding to somebody, it will usually be to make sure that I say something positive or say nothing at all.”—Sheila
	“… always assume that if there is two ways to read something then thinking the kinder way is the way that somebody wants you to read it; I think it is a good rule of thumb because you know like I said, it is hard to interpret tone.”—Anthony
	“So, [an important aspect is] being respectful, you know only saying things that you would say to other people for the most part being particularly I would say from a department account you know being very positive about all of the people that you work with. I think kind of from a formal account, really you probably have to be positive, [an] uplifting voice.”—Piper
Avoid arguments	“I am always surprised at how argumentative some people get. And I think that is a little bit of a shame because I don’t think that … sort of reflects well and this idea about somewhere in between you know maintaining some appropriate composure versus being a skeptic and questioning things. And there are definitely some people who do a good job of that and some people that don’t.”—Sheila
Knowing when to end a conversation	“If there are people that are engaging that seem to have a substantial agenda, then I am more likely to not continue the conversation for long while still being respectful and just [stop] interacting.”—Trevor
Anticipate trolls	“I mean you will occasionally get trolled by negative people…I thankfully haven’t had too much with that but every once in a while, something that I put it out on #1, some naysayer will put something negative or sort of like oh it is just like #6 to do something like this.”—Paula
	“You know there [are] always trolls, right? But I think of one, so before I hit publish on anything, I am like super critical of myself first. So, I think if you already are highly concerned about the words that you use and the product that you are publishing then you are going to find that most people are not out there to be obstinate and/or aggressively negative. And if there is a question then usually it is raised with a more honest and um, straightforward inquiry rather than being malicious.”—Harold
Engage across silos	“I tried to actively engage others across multiple specialties and disciplines. So not just emergency physicians but other physicians, and not just physicians but nurses and technologists and the public. So, it is mostly I think the fact that I tried to cross barriers that might otherwise limit the scope of other people who are on social media.”—Roger
Amplify others	“if there was someone that I know that is doing something cool or having something awesome to happen then I might favorite that or retweet that.”—Trevor
	“I tried to disseminate most of the work that we publish. I try to, anything that we publish that I think is worth making people aware of, I will put a plug in for it. Sometimes I will do a Twitter thread if it is a particularly important study. And I will often tag junior investigators or colleagues to increase their follower count.”—Nadir

### Key themes

Overall, there were several good practices that were felt to be important. Specifically, the domains that our good practice tenets fell within included: 1) Understanding the nuances of specific platforms; 2) Social media team management; 3) Online engagement strategies; 4) Techniques of effective knowledge sharing; 5) e-Professionalism; 6) Potential pitfalls. The following sections detail the perspectives of the various participants, who are named by their randomly selected aliases.

#### 1) Understanding the nuances of specific platforms

One aspect of good practices was knowing and harnessing the specific platforms available within the social media space for effective engagement. Understanding the strengths of various platforms was deemed to be of critical importance across all the participants. Tab. 1 depicts some key platforms that were mentioned and clarifying examples are provided when possible. One participant (Edward) put it well when he described that each of the various media have their own temporal properties: “*They each play a different role … It depends on what the ultimate goal of the interaction is. Certainly, Twitter has much more frequent interaction. The blog is, you know, a weekly thing as is the podcast and then YouTube might be a monthly thing*”.

#### 2) Social media team management

Although some participants retained single-person access to social media accounts (usually around their personal accounts), the preferred mode of conduct for group or institutional social media accounts was to enable shared access across multiple users. The rationale for this was that shared access connoted shared accountability, which made the work lighter for any one person. This finding was also true for blogs and podcasts. Although some of our participants still engaged in single-person blogging and podcasting, many have involved bigger teams. Some even saw these as opportunities to engage in teaching trainees. Harold, for instance, involves residents in his process:“ … *[M]y residents* … *come up with topics* … *So, that is much more of a group collaboration fashion and we* *will* … *edit* *the* *script and* *figure out you know what the teaching points should be …”*

#### 3) Online engagement strategies

We found a number of key engagement strategies that were mentioned by our participants as crucial for building online engagement: culture setting strategies, tailoring the message, responding and responsiveness, and heeding rules of online engagement.

##### Culture setting strategies

Some of the key engagement strategies mentioned by the group were tied to specific platforms or tactics, but others were more generic. Generally speaking, some participants thought that creating an open, welcoming environment was crucial to engagement. One participant, Grace, stated:

*I try and really welcome people. And make sure that when engaging them and there is somebody new in a social media environment, um, synchronous or asynchronous discussion, I make sure to welcome that person … I try and think about netiquette [sic]. And helping people feel successful when they are using social media …*

For some, creating a culture also meant monitoring the quality of online discussions (especially ones they were engaging within) and getting involved when necessary to halt or modify conversations, or to actively avoid frank arguments. For others, this means setting a positive tone with the hopes of actively creating a productive space for sharing. One participant (Piper) noted that “*… from [an institutional] account really you probably have to be positive, [an] uplifting voice*”.

Finally, one other act of culture building identified by our participants was the need to teach and mentor others in this space. Culture building was thought to be collaborative, by encouraging faculty and trainees to engage together via social media for education. Some participants used their podcast or blog as a platform for engaging trainees, apprenticing them into this world while creating new content.

##### Tailoring the message

Our participants thought that social media messages should be tailored (language level, style) to the audience, as Nadir states:

*I have a sense of who I want to read it. And so, if I want the general public to read it then I minimize the jargon and I make it sort of you know interesting to people who are non-medical. But if I want people in my specialty to read it then I don’t mind getting extremely technical*.

##### Responding and responsiveness

Other keys to engagement were thought to be around being responsive to others. From one participant’s point of view (Trevor) it was crucial as an investigator on Twitter to interact with those who sought you out. He stated: “*I don’t necessarily think delving into in-depth articles on Twitter is necessary or appropriate … however, responding to people who are having questions or being critical of things I think is a very reasonable way to go*”. Meanwhile on the receiving end of such engagement, others certainly found that this type of interaction was helpful to themselves as scientists as well.

Responsiveness was thought to have its dark side as well. As you engaged more, our participants highlighted the need to know when to stop having a conversation too. For some, it simply meant halting engagement. For others, they thought it was important to think twice before responding. Taking the emotion out of a disagreement was one strategy highlighted by one of our participants (Jason). He highlights his own strategy for dealing with disagreements online:

I tend to not respond emotively [sic] to anything. If I like something, I will like it. Or I may put a fairly neutral response like: “we have done something similar have a look at this paper”. Rather than saying “you know you’re wrong and we are right for these reasons …”’

##### Heeding rules of online engagement

Many of the other keys to interaction revolved around some rules on using common sense to engage in respectful and positive conversations, while also understanding your goals/intentions for communicating. These rules for engagement are summarized in Tab. 2, alongside explanatory quotes.

#### 4) Techniques for effective knowledge sharing

Participants made note that creating a digital home base such as a website was thought to be of great importance, but largely this was thought to be insufficient for effective knowledge sharing. Social media sharing of new research was thought to be best if it was multimodal, in order to be most useful in promoting that knowledge to the end-users. But simply creating a website and sharing was not thought to be sufficient. There was a perceived need to ‘repackage’ content in a way that was engaging within a specific medium. Bearing in mind the audiences in the social media space, participants like Anthony noted that the role of a good teacher or translationalist in this space is to “*… make [core concepts] easily understandable, accessible, you know put my own little spin and little pearls …*”*.*

Julie noted that while repackaging was of importance, it was crucial to be knowledgeable about end-users. She said that for her podcast, she and her co-host try their best to bear in mind their listenership. She stated:

… *we try to make sure that we explain sort of the basics instead of just assuming because we know we have a lot of medical student listeners and a lot of early resident listeners, in addition to career emergency physicians. And so, we’ve tried to cover the gamut by covering stuff that is interesting to us as … docs but then also to go into a little bit of the sort of basics to an extent, to kind of address the [other audience groups]*.

Some participants also highlighted the need to both produce and selectively amplify high-quality, accurate content. This responsibility was best stated by Anthony who said how crucial it is for producers to be “*just making sure that whatever content you put out, that its quality is accurate, is important as well*”. In an effort to ensure the quality of any educational or knowledge translation projects they undertake, some individuals create networks of trusted advisors and reviewers to review their work prior to publication, an *ad hoc* version of pre-publication peer review, which may or may not be subjected to further scrutiny by editorial members of a social media outlet. Piper stated: “*… if it is at all anything controversial, I usually send it along to a trusted mentor or a friend to read and give me their thoughts on. And then it goes to the editorial board*”.

##### Sharing one’s own research

Good practices for disseminating one’s own research included ensuring that various social media promotional activities were all aligned with the area of research or scholarly interests. Participants highlighted that it is useful to consider integrating alternative media into the knowledge translation process. Specifically, infographics were thought to be of importance in this area, either as a post-publication dissemination technique or directly into research papers so as to facilitate social sharing of the paper by others. Creating a planned and integrated strategy for disseminating a paper after it is published can include alternative and creative forms of expression including infographics, as Piper vividly describes:

… *the other kind of variety of content that I seem to be producing most of these days is like infographics for translation of our research findings … [infographic creation] requires you to do is really distil down what this big study is about … really, it gets you thinking: ‘what is the impact of what we have done?’ … ‘[H]ow do I want to share and frame that for people?’ So, when I do those it ends up being a bit of a self-reflective process on my paper or my research and what it is bringing to the community.*

The challenges of infographics of course are that the visual medium requires a different type of thinking and careful design is important so as not to over-simplify or water down the content. Julie stated: “*… for creating the visual abstracts, I create those for the lay emergency clinician who does not understand large relative risks or odds ratios … And then I tried to convey the results in an as succinct … way that I can*”.

#### 5) e-Professionalism

Similarly, for those who inhabit the online space more as ‘translationalists’ and teachers, the space is similarly riddled with traps. As stated previously, it is imperative for professionals (e.g. physicians and nurses) engaging in online education to be wary of their professional obligations, and all individuals should be aware of how easy it is to unintentionally breach confidentiality. As Harold points out, training in this area is a must: “*We, you know, are very cautious with our residents and faculty and making sure that we are training everybody that you know you are not publishing things that are sensitive material or patient information and all of the common pitfalls that we have seen*”. And even then, educators in the online space should be continually vigilant in assisting those new to the field since, Brandon remarked, “*… there is, you know, potential for … even unintentional patient privacy violations … which again can get people into trouble with their home institutions*”.

#### 6) Potential pitfalls

Several notes of caution were noted within our participant’s interviews, which we grouped into two broad categories: 1) Caveat emptor and the need for criticality with resources; 2) Common pitfalls when broadcasting one’s self.

##### Caveat emptor: The need for increased rigor and criticality for social media resources

Specifically, some participants felt that it was important for users of social media resources to note that their work was not a comprehensive resource, and that they (as the sellers of the evidence) could not replace primary literature or textbooks. Participants highlighted the value of social media to *curate* or *highlight* important resources, while also recognizing that sometimes social media will highlight sensationalist (and less rigorous) resources at times. Trevor noted: “*I don’t believe that the use of social media replaces any of my academic reading*”. Specifically, Trevor noted that it was important to make clear to users that social media-based resources should not supplant the use of the primary scientific literature. This was similarly mirrored by other comments who cautioned those in this space to recognize their responsibility for fact-checking and ensuring accuracy of online content prior to distributing it.

##### Common pitfalls when broadcasting one’s self

When engaging in disseminating one’s own work on social media, one must be aware that others may not perceive this as a simple act of sharing. As one participant (Piper) highlighted, there was a fine line that needed to be walked between ‘bragging’ and disseminating your content. This quote highlights her perspective:

*You know I think the biggest balance is… towing this line between self-promotion and sharing your resource or sharing your research and getting stories out about your studies and about what you think is important … I don’t want to be seen as pushing it at people aggressively … I also want to be seen as humble and thoughtful.*

Many of our participants felt the weight of responsibility upon them when speaking about their role as an openly identifiable online physician. Examine this one statement by a participant (Darcy):

… *[T]here is a … reason that the Doctor Oz show is the Doctor Oz [show], and it is not just [titled] talking about some shit [sic] with a guy named Bennett. I mean, there is gravitas. There is a professionalism to being a physician. … [there], is a factor of credibility because anything that I put out there I sign my name to.*

## Discussion

**Fig. 1:**
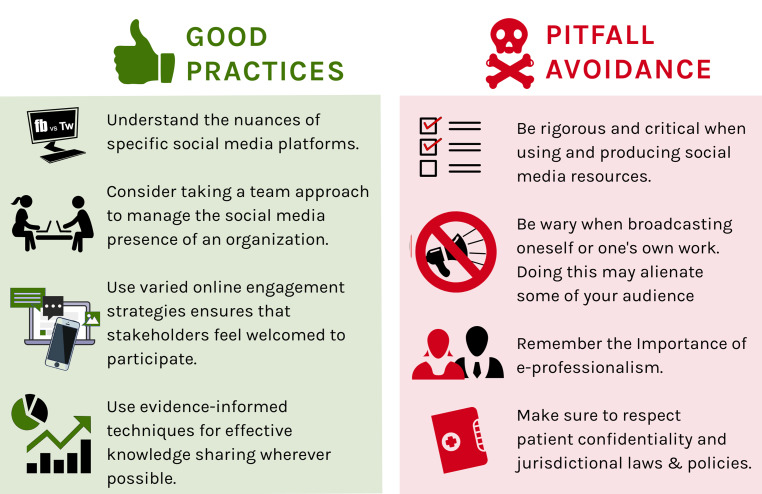
A summary of our study’s themes around key considerations in the use of academic social media

In this study we have sought the insights of peer-identified influencers and leaders within the social media learning environment to understand good practices and potential pitfalls for those entering this space. Through their aggregate experiences, several key themes are summarized in Fig. 1 as key takeaways for our readers.

Our findings should be interesting to both new scholars, who are seeking to carve their niche within the academy, but also those seeking to foster others’ success by capitalizing on social media as a platform for dissemination. There is an evolving role for new scholars in today’s academic milieu who can help with the translation of knowledge as teachers (translational teachers), and those who effectively engage their target audiences and key stakeholders via social media as scientific investigators (interactive investigators) [5]. This paper provides empirical data which highlight these new roles, and how they are increasingly sophisticated. Participants who identify with these roles have a growing mandate to organize and add structure to a zone where social media meets academia. This aligns with the structuralist phase of the greater Free Open Access Medical education (FOAM) movement [14], which includes new roles such as Social Media editors for journals [15].

Our participants also explained how they are not solely translational teachers, but at times must wade into promoting their own scientific or scholarly work, which shifts their role and requires new considerations. As critical clinicians, they saw the need to be actively skeptical of the science and participate in scholarly discourse around science that is published in any format—whether it be in a high-impact journal or a high-traffic blog. As teachers, our participants reflected upon how they seek to produce high-quality content and to educate others to appraise content in the social media space. Finally, as either interactive investigators or translational teachers, they remarked on their sense of responsibility around the need to be accurate and not fall into the trap of becoming a ‘celebrity’ or ‘science Kardashian’ [16–18]. Due to their professional identity, our participants found it imperative to consider content accuracy and saw themselves as accountable for ensuring the validity and veracity of their content. Similarly, e‑professionalism was found as a thread throughout the interviews; the themes found in our present study were similar to work that has been done on e‑professionalism within medical student and trainee populations [19–22]. That said, compared with prior literature [19, 22] which largely focused on the hazards of social media towards professionalism, our participants heavily de-emphasized this concept, relegating it to a concept that must be incorporated but not in the front of their minds.

Many of our findings show the parallel between our participants’ social media use and the techniques used by modern marketing strategists to gain attention; for example, repackaging content in various formats to suit consumers (e.g. infographics, podcasts, easy-to-read blogposts) and adequate brand alignment between content producers and target audiences were identified as essential practices. Many of the concepts discussed by Davenport and Beck [4] have a suitable mapping to the online engagement strategies.

As Davenport and Beck write in their book, those that: “… succeed in the future will be those experts not in the time management, but in the *attention management*” [4]. Phenomena such as infographics and visual abstracts are tightly associated with tactics that would help grab learners’ attention and prevent the TL;DR (“too long; didn’t read”) label that is dreaded in the social media world [23].

Translating longer articles to *capture attention* is a phenomenon that aligns very closely to the existence of an attention economy within social media-based knowledge translation and medical education. Tailoring the message to your target audience helps you to capture voluntary attention through the effective structuring and design of your content. Being responsive and responding well to others also help our participants to engage the *attractive attention*—reinforcing and providing positive feedback to those who engage with your material. Heeding rules of online engagement to avoid unwarranted negative reactions for violating the cultural norms is closely connected to the concept of avoiding *aversive attention*. Our participants also found it harder to walk the fine line between self-promotion and knowledge translation/dissemination, worrying about how their peers in the profession might view them. Many health professions education researchers and scholars may find that this resonates with them as well. Meanwhile, some types of attention that Davenport and Beck identify in their work go beyond the depths of what we found currently within the responses of our respondents. For example, our participants did not specifically speak to the value of creating an academic brand to capture the *back-of-mind attention *that Davenport and Beck describe; however, there is increasing discussion around this in academic medicine [24].

Our present study has a number of limitations. First off, our lead investigator is fairly immersed in the world of online education and knowledge translation, and this may have affected our interpretation of the participants’ words. To optimize her distance from the actual respondents, we ensured that she did not interview any of the participants. We also involved the research assistants and transcriptionist in redacting the individual transcripts to ensure that she was not privy to the identity of the various participants. In the analysis phase, we used multiple strategies to ensure the rigor of our analysis, acknowledging that our lead investigator brought with her both her ‘insider’ expertise, affording us a unique perspective on these topics.

### The future of #MedEd in social media

Going forward, the evolution of thinking within the social media-based education space will likely become increasingly aligned with the thinking of the participants within our study. We foresee issues around ensuring that we grab the attention (as well as the hearts and minds) of our audiences in social media; this will be of growing importance going forward. With the rise of a generation of physicians who essentially grew up with social media, we will gradually see the integration of these platforms into our scientific and educational circles [25, 26]. And while we cannot generalize across a whole generation [27], it is clear that the global increase in usage and popularity of social media as a major communication platform provides concrete evidence of the changes in the way we communicate to learners and colleagues [1, 2, 28]. The depth of considerations and thinking on the various topics around safe social media utilization was coupled with an ease with which some of the participants understood how to best harness the power of these new media.

## Conclusions

By engaging leaders and early adopters of social media as a tool for scientific discourse, knowledge translation and education, our work identifies good practices to guide health professionals and other stakeholders in a space that is rapidly growing in both pervasiveness as well as importance. Key strategies revolved around content delivery, audience engagement, and e‑professionalism as well as being critical of one’s own accuracy and role on social media.

## Caption Electronic Supplementary Material

Our study’s interview guide is supplied in this appendix.

Supplemental Table 1: Demographics of participants

Supplemental Table 2: Social media platforms used by participants
