# Supplementary material for: Good practices in harnessing social media for scholarly discourse, knowledge translation, and education
Source: Perspect Med Educ. 2020 Aug 20;10(1):23–32. doi: 10.1007/s40037-020-00613-0 (PMC7439800; doi:10.1007/s40037-020-00613-0)
Supplement: Supplementary file 1 — Our study’s interview guide is supplied in this appendix. [file 40037_2020_613_MOESM1_ESM.docx]

**INTERVIEW GUIDE – PART A**

**STUDY TITLE**: Social Media and Knowledge Translation: A Qualitative Study

**PRINCIPAL INVESTIGATOR**: Dr. Teresa Chan, MD, Department of Medicine at McMaster University, Hamilton, Ontario, Canada, [teresa.chan@medportal.ca](mailto:teresa.chan@medportal.ca)

**CO-INVESTIGATORS**: Priya Thomas, Emma Bridgwater, Mark Lee, Aisha Mohamed

**INVESTIGATION SITE**: McMaster University

**TIME COMMITMENT FOR PART A**: The interview will run for approximately 30 minutes.

**INTERVIEW GUIDE**

**PART 1: Introduction**

*Note: The facilitator of the phone interviews will use this as a script.*

My name is _____ and I will be the research team member conducting this interview. We are looking to understand how and why scientists and clinicians engage in social media for the purposes of teaching, translating, and impacting others. We hope that this will inform a best practice guide that can help to instruct others on how to do this well.

The interview will last approximately 30 minutes. Any information shared by you during this interview will be considered strictly confidential; only de-identified data will be analyzed or shared. This conversation will be audiotaped and transcribed. In the transcription all individual identification will be deidentified.

**PART 2: Consenting Process**

Prior to this interview, you were sent an information sheet on the nature of this study.

Do you have any questions or concerns related to your consent to participation in this study?

This interview will be recorded. Your consent will be captured through this recording. Do you consent to participate?

**Yes/No**

Thank you. The date and time of your consent will be recorded for our records.

**PART 3: Research Study Questions**

| **INTRODUCTION** | | |
| --- | --- | --- |
| **Q#** | **Question** | **Prompts** |
| Q1  **[G]** | Can you describe your current academic status (e.g. rank) and position within your organization? How does social media relate to this role? | *What does your role actually entail from day to day?*  *Can you list any places where you use social media to enhance your role?* |
| Q2  **[G]** | What social media platforms do you regularly engage in? | *What defines a “SM” platform varies:*   - *NA it tends to be Twitter/FB* - *Africa sees heavy usage of What’s App* - *Personal blogs* - *Podcasts*   *Why this platform?*  *If multiple platforms, why? How do you manage everything?*  *Do you engage in different platforms for “personal” vs. “professional” use? What drives that choice?* |
| Q3  **[G]** | What do you see your role on social media as? | *What typical activities do you engage in when using social media? Is there a theme/common thread to your typical activities?*   - *Ex. Do you post a lot of work from your own lab?* - *Sharing/critiquing of guidelines within a social group?* - *Manage the social media presence for a group (ex. hospital/university department)*   *Do you consume content more than you produce content?* |
| Q4  **[G]** | Given the following descriptions, which of these roles would you say is the best descriptor of you?  Do you feel you fit into more than one? |  |
|  | **Translational Teacher:** Strong, often trained, educators; they work with researchers to help with knowledge translation and getting the word out about new studies/findings. *(the PR)*  **Critical Clinician**: Skilled at critical appraisal, these individuals critique and analyze new studies/findings in an open forum. *(The restaurant critics)*  **Interactive Investigator**: Produce new studies/findings, while engaging with end-users to explain and receive feedback for improving their research. *(The traditional scientist)*  **Skeptics:** Are openly critical of, and/or dislike FOAMed and the surge in social media as a method of rapid knowledge translation for a variety of reasons.  **Agnostics:** While they engage in social media, these individuals typically have no strong opinion on FOAMed itself, positive or negative. | |

| **GENERAL QUESTIONS FOR ALL IDENTITIES** | | |
| --- | --- | --- |
| **Q#** | **Question** | **Prompts** |
| Q4  **[G]** | What got you started using social media? | - *Have you always had a strong online presence?* - *Personal enjoyment/fulfilment?* - *Some people see it as a good career move/way to stay current in their field* - *Was it an institutional requirement/expectation?* - *Have you changed the platforms you use?* |
| Q5  **[G]** | How much time do you spend in a given week producing content?  How much time do you spend engaging with FOAMed produced by other users in a week? | *Posting, Tweeting, messaging in the group, etc.*  *When? (ex. before work, throughout the day, weekends)*   - *Do you set aside specific time to engage in this?*   *Do you find yourself engaging in less academic reading (ex. journals, etc.) as a result?* |
| Q6  **[G]** | What “best practices” do you employ when engaging with social media/your audience? | *How do you decide what posts to retweet/share/promote?*  *How do you ensure the “quality” of the things you share/post?*  *How do you respond to critics online?*  *What do you do to maintain a “professional” identity? Do you?* |
| Q7  **[G]** | Has your social media/online identity ever changed? Why? | *Some people start out engaging online with a more “personal” identity and have shifted to a more professional one over time.*  *Shift may be prompted by necessity (ex. running an institutional profile), or simply because it is easier.* |
| Q8  **[G]** | Do you ever feel like your identity isn’t “you”?  If so, how do you keep your professional and personal identities separate?  What strategies do you use to disengage from your personal identity when posting in a professional capacity? | *Especially relevant for those individuals managing accounts for 3^rd^ parties.*  *Do you find this to be difficult? Have these identities ever come into conflict with one another?*  *How has your approach changed over time? (i.e. since you first started having multiple identities, to now)* |
| Q9  **[G]** | How does your institution/department view social media and its role in medicine?  Your colleagues? | *Do you feel supported in your efforts?*  *Do you/have you experienced any direct institutional barriers when getting involved? Any positive encouragement from your institution?*  *Are your social media activities counted as “scholarly activities”? Are you rewarded/remunerated for them? How are they considered?* |
| Q10  **[G]** | How do you feel social media/FOAMed has changed or impacted the field of medicine?  Your field specifically? | *Do you think the impact has been positive or negative?*  *Why?* |
| *From here, begin asking questions specific to the role that we have identified them as; if they have self-identified as more than one, or as a different role than pre-assigned, ask the questions for those as well.* | | |

| **Specific Questions for the Translational Teacher** | | |
| --- | --- | --- |
| **Q#** | **Question** | **Prompts** |
| Q1  **[TT]** | What process do you use when choosing what new work you choose to disseminate? | *Do you work with the same researcher group consistently?*  *How do you find the work that you post/share?* |
| Q2  **[TT]** | How do you approach communicating with the much broader audience that you can reach with social media? | *Everyone from senior physicians, to medical students, to the general public can access information on most social media platforms*   - *How do you navigate communicating effectively with this broad audience?* - *Do you focus your efforts on specific groups?* |
| Q3  **[TT]** | Do you have anything else you would like to explain about your viewpoints? |  |

| **Specific Questions for the Critical Clinician** | | |
| --- | --- | --- |
| **Q#** | **Question** | **Prompts** |
| Q1  **[CC]** | How did you develop your credibility/visibility as a critic on social media when starting out? | *What challenges did you face in trying to increase your online “impact factor”?* |
| Q2  **[CC]** | Why do you engage in open-forum critical appraisal?  What advantages does this have over the more traditional peer-review process that make it attractive for you? Any disadvantages? | *Is it timeline? (traditional methods = typically quite slow)*  *Enjoyment of real time feedback?*  *Disadvantages – ex. communicating ideas properly on platforms with limited character count;* |
| Q3  **[CC]** | Do you have anything else you would like to explain about your viewpoints? |  |

| **Specific Questions for the Interactive Investigator** | | |
| --- | --- | --- |
| **Q#** | **Question** | **Prompts** |
| Q1  **[II]** | Does advocating largely for your own research via social media pose any challenges? | *Is it difficult to balance the role of “investigator” with the role of “PR”?*  *How do you keep these identities separate? Do you?* |
| Q2  **[II]** | Has your involvement with social media, and real-time feedback from end users, changed your approach to research? How? | *Ex. your process for developing questions, how you incorporate feedback into future work, etc.* |
| Q3  **[II]** | Do you have anything else you would like to explain about your viewpoints? |  |

| **Specific Questions for the Skeptic** | | |
| --- | --- | --- |
| **Q#** | **Question** | **Prompts** |
| Q1  **[S]** | What drives you to actively engage in expressing your skepticism via social media? | *Why do you find this time valuable?* |
| Q2  **[S]** | What about FOAMed/the increasing use of social media in medicine and medical research do you find problematic? | *What changes could be implemented, if any, that would improve your outlook?* |
| Q3  **[S]** | Do you have anything else you would like to explain about your viewpoints? |  |

| **Specific Questions for the Agnostics** | | |
| --- | --- | --- |
| **Q#** | **Question** | **Prompts** |
| Q1  **[A]** | What drives you to participate in social media/FOAMed, given your neutral outlook? | *Institutional requirement? Personal enjoyment? Pressure from colleagues?* |
| Q2  **[A]** | How do you stay “neutral”? What aspects of FOAMed keep you from picking a side in what is a fairly controversial debate? | *Just not interested?*  *What pros/cons do you see to the movement? How do you see these balancing out? What might tip your attitude one way or the other?* |
| Q3  **[A]** | Do you have anything else you would like to explain about your viewpoints? |  |

**PART 4: Conclusion**

Question:

Lastly, is there anything else you want to share with me?

Question:

*[ONLY FOR TRANSLATIONAL TEACHERS, INTERACTIVE INVESTIGATORS, AND CRITICAL CLINICIANS]*

We are inviting individuals who are interested in sharing more about their institutional and environmental enablers and barriers regarding social media usage for professional purposes. Would you like to be contacted to continue your discussion regarding this topic? It will take another interview lasting about 30 minutes again that we can arrange at time of your convenience.

**Yes / No**

Thank you for your time and your participation; this has been a very fruitful discussion. If there are additional follow up questions I would like to pursue, do I have your permission to contact you?

If at a later date you would like to add or clarify anything shared during this interview, please contact the local Principle Investigator. Thank you.

Question for all:

Finally, we are using a snowball sampling technique to seek other interviewees. Can you name a person in teach of the following categories that come to mind?

1) Translational Teacher

2) Interactive Investigator

3) Critical Clinician

4) Skeptic/Agnostic

We may touch base with you to help us get in touch with this person by providing us with an email.
