# Supplementary material for: Good practices in harnessing social media for scholarly discourse, knowledge translation, and education
Source: Perspect Med Educ. 2020 Aug 20;10(1):23–32. doi: 10.1007/s40037-020-00613-0 (PMC7439800; doi:10.1007/s40037-020-00613-0)
Supplement: Supplementary file 2 — Supplemental Table 1: Demographics of participants [file 40037_2020_613_MOESM2_ESM.docx]

**Supplemental Table 1:** Demographics of participants

| **Feature** | **Frequency (%)** |
| --- | --- |
| **Gender** | |
| Female | 7 (41%) |
| Male | 10 (59%) |
| **Roles** | |
| Teacher/Educator | 17 (100%) |
| Clinician | 15 (88.2%) |
| Researcher | 13 (75.6%) |
| Academic leader | 7 (42.1%) |
| Implementation specialist | 5 (29.4%) |
| Clinical leader | 3 (17.6%) |
| **Academic qualifications** | |
| MBBS / MD / DO | 10 (58.8%) |
| Master’s degree (MBA, MPH, MSc, etc.) | 11 (64.7%) |
| Doctorate degree (PhD) | 5 (29.4%) |
| Fellowship certification | 10 (58.8%) |
| **Countries of origin** | |
| USA | 8 (47.1%) |
| Canada | 5 (29.4%) |
| Australia | 2 (11.8%) |
| Netherlands | 1 (5.9%) |
| New Zealand | 1 (5.9%) |
| *DO* Doctor of Osteopathy; *PhD* Doctor of Philosophy; *MBA* Masters of Business Administration; *MBBS* Bachelor of Medicine and Bachelor of Surgery; *MD* Medical Doctorate; *MPH* Masters of Public Health; *MSc* Masters of Science; *USA* United States of America | |
