# Supplementary material for: Good practices in harnessing social media for scholarly discourse, knowledge translation, and education
Source: Perspect Med Educ. 2020 Aug 20;10(1):23–32. doi: 10.1007/s40037-020-00613-0 (PMC7439800; doi:10.1007/s40037-020-00613-0)
Supplement: Supplementary file 3 — Supplemental Table 2: Social media platforms used by participants [file 40037_2020_613_MOESM3_ESM.docx]

**Supplemental Table 2:** Social media platforms used by participants

| **Platform** | **Frequency (%)** |
| --- | --- |
| **Social media platforms** | |
| Twitter | 17 (100.0%) |
| Facebook | 8 (47.1%) |
| Slack | 8 (47.1%) |
| LinkedIn | 7 (41.2%) |
| WhatsApp | 6 (35.3%) |
| Instagram | 3 (17.6%) |
| Reddit | 3 (17.6%) |
| Snapchat | 1 (5.9%) |
| **Scholarly social platforms** | |
| Google Scholar | 10 (58.8%) |
| ResearchGate | 8 (47.1%) |
| ORCID Researcher Identifier | 8 (47.1%) |
| Academia.edu | 2 (11.8%) |
